# Case of Internal Strangulation That Led to a Suspicion of Poison

**Published:** 1829-07-01

**Authors:** 


### XLII.

### REMARKABLE CASE OF INTERNAL STRANGULATION, WHICH LED TO A SUSPICION OF POISON, AND A LEGAL INVESTIGATION. By M. ROSTAN, M.D.

The following case is interesting in a pathological as well as in a medico-legal point of view. It created considerable sensation in Paris.

Mademoiselle Hullin, a celebrated opera dancer, aged 28 years, had experienced numerous attacks of abdominal inflammation in the course of her life, and had often undergone rigorous antiphlogistic discipline in consequence. Three years ago her stomach was in such a weak state, that she could not bear the mildest species of food without sickness or pain. In time, however, she recovered from this state, under

M. Dufour, and could use ordinary diet with impunity. In September, 1828, she married the Sieur St. Maurice, and her health continued good till the 17th February, 1829. On that day she was seized with vomiting of glairy matters mixed with her food—extreme restlessness—anxiety—some pain in the abdomen but without heat of skin or acceleration of pulse. In the night these symptoms augmented—a physician was called in, and prescribed leeches to the abdomen. *18th February.* Extreme restlessness—faintness — propensity to make water and go to stool, but without effect. A lavement only brought away some glairy mucus. Pulse and skin were still natural—no thirst—little or no pain in the abdomen, according to the report of one physician, but great pain according to another. Only cataplasms and fomentations were used this day. At eleven o'clock at night, there was some remission of the symptoms—there was moisture on the skin, and the physicians augured favourably. *19th.* This morning she suddenly complained of violent pain in the right iliac region, which continued till mid-day, when vomiting of yellowish matters came on. The vomiting increased, and the matters thrown up became black in colour, and evidently fæcal. It was then considered that ileus existed. She was put into a warm bath for three hours, but without relief. The pulse became small and quick—the abdomen distended—the features shrunk, and the patient sunk at ten o'clock that night.

The sudden and violent death of this lady gave rise to rumours and suspicions of poisoning, and the husband was accused of the act. The Sieur, conscious of his innocence, demanded the exhumation of the body, and a medico-legal investigation of the death. The examination was made by a physician in the presence of a judge of instruction and some others. He reported a phlogosis of the stomach, redness of the duodenum—incipient sphacelus of the ileum—nothing else remarkable in the body. The physician considered that there was nothing in the dissection which could authorise the suspicion of poison. The tribunals, however, were not satisfied with this result, and Messrs. Orfila and Rostan were ordered to reinspect the parts. They very soon detected the cause of death, namely, a portion of intestine (ileum) strangulated by a small band (bride) of condensed cellular membrane. This band was not more than a quarter of a line in breadth—and could it have been suspected before death, an operation would have readily removed the constricting cause. This little band was about an inch in length, adherent by one extremity to one of the surfaces of the mesentery, and by the other to the opposite surface, thus comprehending and strangling the ileum like a ring on a purse. The cause of the dreadful symptoms and the fatal issue was thus explained, and all suspicion of poison removed, especially as the contents of the stomach and bowels were carefully analyzed.

Our readers will remember the case of Mr. Belzoni's servant, which we published a few years ago, and which very closely resembled the above, except that the poor fellow lingered out twelve or fourteen days—and after all there was no sphacelus of the gut. The patient died from the strangulation and the accumulation of matters above the constriction.—*Archives Generales.*

